# Lung ultrasound findings predict weaning failure from mechanical ventilation

**DOI:** 10.1186/cc13488

**Published:** 2014-03-17

**Authors:** AC Pecanha Antonio, P Souza Castro, LF Schulz, J Maccari, R Oliveira, C Teixeira, M Knorst

**Affiliations:** 1 Hospital Moinhos de Vento, Porto Alegre, Brazil; 2 UFRGS, Porto Alegre, Brazil

## Introduction

Lung ultrasound is increasingly becoming a diagnostic tool in the critical care setting. The B-line is an artifact that correlates with interstitial edema. Decreases in intrathoracic pressure during a spontaneous breathing trial (SBT) will augment venous return and impede left ventricular ejection, increasing intrathoracic blood volume. Therefore, the presence of cardiovascular dysfunction can contribute to weaning failure (WF). A randomized trial concluded that bedside lung ultrasound could predict post-extubation distress through changes in aeration during a T-tube test; however, it could not screen patients before submission to a SBT [1]. We aim to assess the reliability of lung ultrasound as a predictor of weaning outcomes.

## Methods

We conducted a prospective, multicenter, observational study in two adult medical-surgical ICUs. Lung ultrasound was performed immediately before SBT. Three or more B-lines in a single view were called a B-pattern. B-predominance was defined as a B-pattern on at least one of the four anterior chest wall zones. All enrolled patients met eligibility criteria for ventilation liberation. Patients with tracheostomy were excluded.

## Results

During 2 years, 250 SBTs were analyzed. WF, defined as an inability to tolerate a T-tube trial during 30 to 120 minutes, occurred in 51 (20.4%). There was a higher prevalence of chronic obstructive pulmonary disease in the WF group as well as higher duration of mechanical ventilation. WF patients were also younger. Patients succeed at SBT and were extubated at first time in 75.9% of cases. We observed a significant association between B-predominance prior to submission to SBT and WF (OR = 1.99 (1.04 to 3.84)). For diagnosing WF, B-predominance showed 69% sensitivity, 48% specificity, 25% positive predictive value, and 86% negative predictive value.

## Conclusion

The finding of B-predominance at bedside lung ultrasound performed before SBT predicts WF, although it shows low accuracy.
